# Impact of the workforce distribution on the viability of the osteopathic profession in Australia: results from a national survey of registered osteopaths

**DOI:** 10.1186/s12998-018-0204-0

**Published:** 2018-09-11

**Authors:** Amie Steel, Nigel Jackson, Raymond Blaich, Mathew Kirk, Jon Wardle

**Affiliations:** 1 University of Technology Sydney, Faculty of Health, Australian Research Centre in Complementary and Integrative Medicine, Ultimo, NSW Australia; 2 Endeavour College of Natural Health, Office of Research, Brisbane, QLD Australia; 3 Southern Cross University, School of Health and Human Sciences, Lismore, NSW Australia

**Keywords:** Health workforce, Osteopathy, Survey, Workforce sustainability

## Abstract

**Background:**

Workforce distribution has an important influence on the quality of healthcare delivered in a region, primarily because it impacts access to health services in the community and overall health equity in the population. Distribution of osteopaths in Australia does not appear to follow the Australian population with the majority of osteopaths located in Victoria. The implications of this imbalance on the osteopathic workforce have not yet been explored.

**Methods:**

A secondary analysis of data from a survey of 1531 members of Osteopathy Australia in 2013. The analysis focused on the practice and occupational characteristics associated with practice locality.

**Results:**

The survey was completed by a representative sample of 432 osteopaths. Respondents practicing outside Victoria were more likely to report higher income across all income brackets, and were less likely to report a preference for more patients.

**Conclusions:**

The Australian osteopathic profession should examine the issue of imbalanced workforce distribution as a priority. The results of this study are worth considering for all stakeholders as part of a coordinated approach to ensure the ongoing health of the Australian osteopathic workforce.

## Background

Workforce distribution has an important influence on the quality of healthcare delivered in a region, primarily because it impacts access to health services in the community and overall health equity in the population [1]. Imbalances in workforce distribution in Australia has been found to be linked to a number of factors, including undesirable lifestyle and living arrangements, access to professional development opportunities (for both the practitioner and their family), or personal and professional isolation [2]. Within private health services (those reliant on out-of-pocket expenditure rather than public employment), a younger and older practitioner age has been associated with an increased likelihood a health professional would leave their current position compared to mid-age practitioners. This intention to leave is primarily linked to a sense of professional isolation and does not necessarily reflect an intention to leave the profession more generally [3]. Business viability (due to staffing issues, morale and the balance between supply and demand) also has a significant impact on workforce distribution [4]. Imbalanced distribution of health workforce can present challenges to the profession itself when attempting to garner national or state-wide support for new programs and services [5].

### Osteopathic workforce in Australia

Osteopathy is a manual therapy which follows the principle that structure and function are closely integrated by assessing a person’s musculoskeletal, neurological and visceral systems [6]. In Australia, osteopaths are regulated professionals registered through the Australian National Registration and Accreditation Scheme [7]. There is some degree of integration of osteopathy into the Australian public health system, with patients able to access public funding for osteopathic treatments via government agencies such as Medicare [8] and Department of Veteran Affairs [9], and relatively high levels of referral to osteopaths by general practitioners [10].

In 2015 there were 1925 registered osteopaths in Australia. Distribution of osteopaths in Australia does not appear to follow the Australian population. The majority (52%) of Australian osteopaths are based in Victoria (which has less than one quarter of the Australian population) [11]. New South Wales (28%), and Queensland (9%) also have relatively large populations of osteopaths, whilst very few osteopaths are practicing within the other states and territories (< 3% per state/territory) [11]. Currently there are three universities in Australia which offer osteopathy programs accredited by the Australasian Osteopathic Council [12], two of which are located in Victoria (Victoria University and RMIT University) with the third located in New South Wales, in a regional location close to the Queensland border (Southern Cross University). Existing practice research suggests that osteopaths draw an income primarily from treating patients within privately-owned practices with the average income for an osteopath reported to be less than $70,000 per annum [12].

Whilst there is significant imbalance in the workforce distribution of the osteopathic profession in Australia, the implications of this imbalance on the osteopathic workforce have not yet been explored. As such, this paper provides the first examination of the factors which may be linked to osteopathic workforce distribution with a specific focus on the relationship between practice location and both practice income and satisfaction with patient load.

## Methods

### Design

A secondary analysis of data collected by Osteopathy Australia – the professional association for osteopaths in Australia – through a members’ survey was undertaken. The survey was administered in 2013 and permission to access the raw data was granted to the research team in 2015 for the purpose of this analysis.

### Sample

All Australian osteopaths who were practicing members (*n* = 1531) of Osteopathy Australia in 2013 were invited to participate in the survey.

### Instrument

The 75-item survey is administered every 4 years to members of Osteopathy Australia to inform the organisation’s activities. The items were developed by an external survey company with input by the management of Osteopathy Australia and the majority of survey items pertained only to details relevant to Osteopathy Australia’s organizational activities. The instrument covered a range of topics relevant to osteopathic practice of which ten items were included in our analysis including sociodemographic characteristics (including annual income), practice locality, practice characteristics (including fee structures, hours worked per week, duration of treatment sessions), and satisfaction with patient load. Response options were primarily using Likert scales (e.g. Never – Always) or fixed variable responses (e.g. practice location item listed each Australian state). Survey items which allowed open responses included years in practice, patient visits per week (new and ongoing), hours worked per week, fees charged per treatment.

### Analysis

Frequencies and percentages were calculated based on the provided data. Continuous variables related to practice income were converted to categorical variables before analysis. Categories were developed to reflect broad clusters of income stratification and to ensure sufficient numbers were included within each category to permit robust analysis. The categorical variable for practice location was collapsed into combined variables where small numbers existed within categories (e.g. South Australia, Queensland, Tasmania, Western Australia, Northern Territory, Australian Capital Territory). This new category was also developed in line with the known geographical maldistribution of osteopaths to ensure that the states with larger numbers remained separate and intended analysis was supported by the numbers in the new category. Representativeness of respondents was determined by chi square tests comparing respondent demographics (gender and geographical location) with data from the Osteopathy Board of Australia [13]. Chi square tests were applied to examine the relationship between categorical variables of interest such as locality, hours worked per week, income and perception of patient load. Analysis of Variance tests (ANOVA) were used to compare between continuous and categorical variables. Linear logistic regression analysis was undertaken to identify the likelihood that practice location impacted upon satisfaction with patient load between osteopaths practicing in Victoria and osteopaths practicing in other states. A new binary variable for practice location was generated to support this analysis (*practicing in Victoria* or *practicing outside of Victoria*). Two different models were applied to independently examine the relationship between practice location and the other two variables of interest. The models were tested for goodness of fit using a chi square test. Stata 14.1 statistical analysis program was used for all analysis.

### Results

The survey was completed by 432 osteopaths (response rate = 28.3%) which is equivalent to 23.6% of all registered osteopaths in Australia. Females represented over half (56.5%, *n* = 244) of the respondents. The majority of practitioners practiced in Victoria (52.1%, *n* = 225) or New South Wales (27.3%, *n* = 118) with one fifth of respondents spread across the remaining 6 states and territories (20.6%, *n* = 89). As shown in Table 1, there is no statistically significant difference in gender (*p* = 0.17) or geographical location (*p* = 0.69) between the survey respondents and the characteristics of registered osteopaths in Australia as provided by the Osteopathy Board of Australia.

**Table 1:** Comparison between characteristics of registered osteopaths in Australian in 2013 and survey respondents

Characteristic	Registered osteopaths*	Survey respondents	*p value*
	% (*n*)	% (*n*)	
Gender			
*Female*	52.8% (963)	56.5% (244)	0.17
*Male*	47.2% (860)	43.5% (188)	
Principal place of practice			
*New South Wales*	28.4% (518)	27.3% (118)	0.69
*Victoria*	52.7% (961)	52.1% (225)	
*Other states*	18.9% (344)	20.6% (89)	

^a^Data extracted from December 2013 Quarterly Registration Osteopathy Workforce Report provided by the Osteopathy Board of Australia [13]

Table 2 lists the statistical relationship between practice income, practice workload and location of practice as determined by the chi square and ANOVA tests. Osteopaths practicing in states outside of Victoria had seen significantly more patients each week. This was most notable amongst new patient numbers (*p* = 0.002) where osteopaths in Victoria saw on average 4.5 new patients per week whereas osteopaths in the remaining states saw just under 10.0 new patients on average each week. The number of ongoing patients per week was significantly lower in Victoria compared to other states (*p* = 0.044). Reported income also differed significantly between practice locations (*p* = 0.003) with 46% of osteopaths in other states reporting an annual income over $100,000. Similarly, osteopaths earning more than $150 k/year were much more common in states outside of NSW and VIC (p = 0.003). Whilst not statistically significant (*p* = 0.06), a preference for a greater patient load was most commonly reported by osteopaths practicing in Victoria (60.0%) and a similar proportion of osteopaths in other states (63.6%) identified as being happy with their ongoing patient load.

**Table 2:** Relationship between practice income/workload and location of practice

Practitioner Characteristics	New South Wales		Victoria		Other States		*p*
Number of patients per week	mean	min, max (SD)	mean	min, max (SD)	mean	min, max (SD)	
*New patients*	6.0	0, 40 (5.4)	4.5	0, 20 (3.2)	9.9	0, 85 (15.0)	0.002
*Ongoing patients*	44.0	5, 230 (35.8)	34.1	0, 170 (21.4)	43.9	0, 220 (40.1)	0.044
Income per annum (AUD)	%	*n*	%	*n*	%	*n*	0.003
*Under $50,000*	21.2	25	32.6	73	21.4	21.4	
*$50-100 K*	38.1	45	43.3	97	32.6	32.6	
*$100-$150 K*	21.2	25	12.1	27	20.2	20.2	
*More than $150 K*	19.5	23	12.1	27	25.8	25.8	
Practitioner satisfaction with patient load							
*Happy with patient load*	50.0	17	40.4	42	63.6	21	0.06
*Would like more patients*	50.0	17	59.6	63	36.4	12	

Table 3 presents the results of the linear logistic regression models examining the likelihood of higher income and patient load satisfaction for osteopaths practicing in other states when compared with osteopaths practicing in Victoria. The model assessing the likelihood of practice location associated with income was statistically significant (*p* < .001). Based on these analyses, osteopaths practicing outside of Victoria were more likely to report higher income from practice across all income brackets ($50-$100 K: OR 1.27; $100 K-$150 K: OR 2.64; >$150 K: OR 2.82) compared to Victorian-based osteopaths. The likelihood of higher income for non-Victorian osteopaths also increased proportional to the income bracket with the highest income being associated with the highest odds ratio. The model assessing the likelihood of practice location associated with practice load satisfaction was also statistically significant (*p* < .001). Osteopaths practicing outside of Victoria were less likely to report a preference for more patients when compared with Victorian-based osteopaths (OR 0.52).

**Table 3:** Income and patient load satisfaction for osteopaths practicing in other states compared to Victoria

	*Osteopaths practicing outside of Victoria (n = 207)*				
	OR	95% CI	*p* value	*β coefficient*	*Model fit (p value)*
Income (AUD per year)					
Under $50,000	1.00	1.00	–	–	<.0001
$50 – 100 K	1.27	0.78–2.05	0.337	0.236	
$100-$150 K	2.64	1.43–4.86	0.002	0.971	
More than $150 K	2.82	1.54–5.18	0.001	1.039	
Practitioner perceptions of patient load					
Happy with number of patients or would like less	1.00	1.00	–	–	<.0001
Would like more patients	0.52	0.28–0.96	0.038	−0.660	

## Discussion

This paper reports the first analysis of factors associated with the distribution of the osteopathy workforce in Australia. Our analysis highlights a number of key findings which will assist health workforce planning in the Australian osteopathic profession. Firstly, our results show that there is an association between osteopaths’ reported income and the state where they practice. This finding raises important questions about the reasons osteopaths are not relocating to areas with higher income potential. Increased remuneration has been identified as a possible incentive for health professionals to relocate, but it needs to be reflective of both the expenses of relocation and the increased scope practitioners are required to take on in underserved areas [14]. For example, wages not matching the actual job description can create barriers to attracting skilled health workers to work in rural locations, even when wages are otherwise higher than urban locations [14]. Conversely, a recent review of ongoing research suggests financial incentives are not consistently seen to motivate health workers to relocate to rural and remote areas [15]. However, the workforce distribution imbalance for osteopathy in Australia extends beyond low numbers in rural and remote location and as such the transferability of previous research to this particular context may be limited and additional research focusing specifically on the osteopathic profession in Australia is warranted.

Based on our study, the link between income and location of practice may be due to the greater number of patients seen by practitioners in some states. While this finding may be adding to practitioner income, this higher patient load may have negative effects on the workforce sustainability in some locations. The majority of health research associated with patient load focuses on staffing ratios with an overburdening of the health workforce particularly in professions such as nursing [16–18] and the accompanying risk of job burnout [18]. Research into other allied health practitioner groups in rural NSW [14] suggests that unmanageable workloads and a lack of employment opportunities for spouses discourages work in rural areas, even when higher demand for services may mean that the potential for larger practices exists [19]. However, our study highlights a complex relationship in which respondents were dissatisfied with patient load if it was too high or too low. Higher patient load was reported more commonly in areas outside of Victoria, and it is quite likely that osteopaths in these areas are as much at risk of job burnout as other healthcare workers [18]. As osteopathic practice primarily occurs in private clinics, low patient load would also impact on the financial viability of the clinic and as such equally affect osteopathic workforce retention. Given the relationship between locality of osteopathic practice and satisfaction with workload, a more consistent distribution of the workforce would potentially have a positive impact on long term retention of osteopaths in the Australian workforce. Additional research is needed, however, to verify practitioner experience of burnout in these locations as well as explore the drivers that influence burnout in high risk populations. In particular, a closer examination of the patient load threshold which is linked with practitioners’ experience of burnout, and the associated relationship between patient load and the practitioner to population ratio within the community, would prove useful to inform workforce policy.

Our results also align with previous data [13] in which the practitioner community is highest in Victoria, irrespective of the lower income of osteopaths practicing in this locality. One factor to consider in relation to this, is the location and nature of osteopathic training. At present there are only three universities with accredited osteopathic training programs in Australia, two in Victoria and one in regional New South Wales [20], and these localities align with the areas of highest osteopathic workforce concentration. Some professions strategically recruit students who are more likely to practice in underserved areas or support university training in regional areas. Studies have suggested that regional university training of pharmacists – or recruitment of students from regional areas - was a major driver of increasing the pharmacy workforce in these locations [21]. Other research has emphasised the value of including a focus in the curriculum which supports students moving to under-resourced or rural areas after graduation to practice [22]. Key initiatives in these efforts include: offering regional placements; developing proficiency in regional and rural health care through specific course content; and recruiting students from a rural background [22]. The Australian osteopathic university sector may need to develop similar specific strategies and learn from the experiences of other health professions.

This is the first study to examine factors underlying the imbalance in the distribution of the osteopathic workforce and as such there may also be other factors not identified through our analysis which are specific to the osteopathic profession. Chiropractic, for example, does not appear to have the same issues with imbalanced workforce distribution despite facing similar practice environments and training institutions that were historically limited to a few locations [23, 24]. Further research is necessary to determine the specific factors that are influencing workforce distribution among osteopaths, and which factors may be specific to the profession. Alternatively, as the issue of workforce distribution and regional comparison of osteopathic practice characteristics has not been the topic of much critical or rigorous examination - particularly in comparison to other allied health professions - osteopaths may not be aware of the difference in income based on location. More research is needed to better understand this finding. Further studies should examine the factors specific to osteopathy that appear to be influencing greater imbalance in the distribution of workforce in this profession compared to others.

While this study provides the first examination of the occupational factors which may be influencing workforce distribution of osteopaths in Australia, it is not without its limitations. The data was drawn from a secondary analysis of a centrally-administered survey of members of Osteopathy Australia (previously the Australian Osteopathic Association) and as such our analysis may be at risk of sampling bias. However, as Osteopathy Australia membership includes approximately 85% of osteopaths in Australia this is not expected to substantially impact on the findings, particularly given our analysis confirms national demographic representativeness. Furthermore, the data may be affected by responder bias as the survey was completed by self-report. Despite these limitations, the insights afforded by this analysis have the potential to be useful to efforts to enhance the viability and sustainability of the Australian osteopathic profession.

## Conclusion

The Australian osteopathic profession remains clustered around Victoria, even though patient load, income and practice satisfaction is higher in all areas outside that state, and greater growth in osteopathic utilization is observed in other states. Such imbalanced workforce distribution not only means that osteopathic practitioners are not being fully utilized to their capacity to meet Australia’s health needs, but also raises concerns for the progression and sustainability of the Australian osteopathic profession. Examination of the issue of imbalanced workforce distribution should be a priority for the Australian osteopathic profession. The results of this study are worth considering for all stakeholders as part of a coordinated approach to ensure the ongoing health of the Australian osteopathic workforce.
